# Acute onset of orofacial dystonia from promethazine treatment: A case report

**DOI:** 10.1097/MD.0000000000017675

**Published:** 2019-10-25

**Authors:** Ruili Zhang, Jianbo Lai, Jinwen Huang

**Affiliations:** a Department of Psychiatry, First Affiliated Hospital, Zhejiang University School of Medicine; b The Key Laboratory of Mental Disorder's Management of Zhejiang Province; c Brain Research Institute of Zhejiang University, Hangzhou, China.

**Keywords:** acute dystonia, Botulinum toxin, promethazine

## Abstract

**Rationale:**

Promethazine is an antihistamine agent used commonly for nausea and allergy. Along with its anticholinergic and antidopaminergic functions, promethazine is also used for psychiatric symptoms, such as troubling sleep, anxiety, and agitation. Previous studies have reported that promethazine may occasionally elicit acute dystonia in some individuals, especially for young children and pregnant women.

**Patient concerns:**

The 68-year-old female patient was admitted to our hospital because of feeling anxious and intermittent palpitation for over 1 year. She developed acute orofacial dystonia following promethazine treatment.

**Diagnoses:**

Her diagnoses was generalized anxiety disorder.

**Interventions:**

Discontinuation of the offending agent, promethazine, and injection of Botulinum toxin.

**Outcomes:**

The acute orofacial dystonia was finally alleviated by local injection of Botulinum toxin.

**Lessons:**

Careful assessment of the risk of developing acute dystonia is also needed in old patients when initiating the promethazine treatment.

## 1 Introduction

Promethazine, a first-generation antihistamine, is commonly used to treat nausea, allergy, and psychiatric conditions, such as troubling sleep, anxiety, and agitation, due to its sedative function. In addition to a strong antagonist of the H1 receptor, promethazine is also a moderate anticholinergic and antidopaminergic agent.^[1,2]^ These pharmacological characteristics made promethazine potentially to cause movement disorders, such as tardive dyskinesia, akathisia, and dystonia.

Previous studies have reported in children or pregnant women that promethazine is possibly to elicit acute dystonia.^[3,4]^ In a woman taking promethazine (25 mg 3 times daily) for controlling hyperemesis gravidarum, the acute orofacial dystonic reaction occurred within 3 days after the treatment was initiated.^[5]^ Other antiemetics, such as metoclopramide and prochlorperazine, can also lead to an acute dystonic reaction.^[6]^ Compared with metoclopramide, promethazine had a lower rate to cause dystonia when used as intravenous antiemetic therapy.^[3]^ Herein, we present a case of acute onset of orofacial dystonia due to promethazine therapy in an old Chinese woman.

## 2 Case presentation

A 68-year-old woman was admitted to the Department of Psychiatry in our hospital because of feeling anxious and intermittent palpitation for over 1 year. In the very beginning, the patient became anxious, upset, and fearful without any known reasons. Meanwhile, she also repetitively felt chest distress, palpitation, and dizziness. Her daily life was severely disturbed, as she lost her interest in square dancing and had poor appetite and sleep quality. She went to a local hospital and was diagnosed with generalized anxiety disorder. She was prescribed with flupentixol−melitracen (0.5 mg:10 mg in 1 tablet, 3 times daily), paroxetine (20 mg daily), and buspirone (10 mg, three times daily). Her symptoms significantly improved after taking these medications for half a month. However, this patient then stopped these medications on her own because she perceived herself as well. She soon began to feel uncomfortable again and resumed the treatment, but it did not work as satisfying as before. Consequently, her medications were adjusted to venlafaxine (75 mg daily) with paroxetine and buspirone unchanged. But her symptoms did not improve. One week before admission, the patient stopped venlafaxine on her own and reused flupentixol−melitracen (1 tablet, 3 times daily), but it also did not help.

On admission, her previous history of illnesses was carefully reviewed. She had hypertension for over 20 years and took amlodipine besylate tablet (5 mg daily) to stabilize blood pressure. She received partial thyroidectomy 10 years ago and took levothyroxine (62.5 μg daily). She denied any history of smoking or drinking or any other illicit substance abuse. Physical examinations did not indicate any abnormal sign. Routine blood test, liver and renal functions, thyroid profiles, tumor markers, and infectious indictors were all normal. Electrocardiography, echocardiography, and chest computed tomography were also normal. Cranial magnetic resonance imaging revealed ischemic lesions in bilateral basal ganglia and periventricular areas. She was again diagnosed with generalized anxiety disorder. Flupentixol−melitracen (1 tablet, 3 times daily) was all discontinued and the dose of paroxetine was increased to 30 mg daily. In addition, a fixed-dose combination of promethazine−chlordiazepoxide tablet (1.25 mg:1.25 mg in 1 tablet, 3 times daily) was added to help control anxiety and aid sleep. However, the 2nd day after taking the promethazine−chlordiazepoxide tablet, this patient developed acute dystonia, which presented as involuntary orofacial movements with slurring speech. Considered as the offending agent, promethazine−chlordiazepoxide was discontinued. Mecobalamine, vitamin E, and clonazepam were added, but the condition did not improve. Paroxetine was then also discontinued and sertraline was initiated and titrated up to 100 mg daily. Neurological consultation agreed with the diagnosis of acute dystonia and recommended the use of Botulinum toxin. Two months after the onset of orofacial dystonia, the condition was still not alleviated. The patient received injection of Botulinum toxin and the symptoms significantly improved.

The Hospital Ethical Committee approved this case study. The patient and her daughter had delivered written informed consent for publication of this case.

## 3 Discussion

Neuroleptic-induced dystonia usually occurs when the offending drug is initiated or the dosage is increased. We report a case of acute orofacial dystonia in an old woman, who started promethazine−chlordiazepoxide treatment for only 1 day. The dystonia persisted for 2 months till the injection of Botulinum toxin.

An acute dystonia reaction can occur after exposure to any domapine receptor antagonist, characterized by involuntary contractions of muscles in different body parts. The abnormal movements or postures may present in a sustained or intermittent, reversible or irreversible manner. It has reported that 50% of the acute dystonia caused by domapine receptor antagonist occurs with 2 days and 90% occurs within 5 days of initiating the treatment.^[7]^ An imbalance of the dopaminergic and cholinergic systems in the basal ganglia is thought to be the major pathophysiological etiology of acute dystonia.^[8]^ Other researchers also proposed the involvement of the antihistaminic and GABA-ergic systems.^[5]^ Male gender, young age, previous history of acute dystonia, and cocaine use are possible risk factors for acute dystonic reaction.^[9,10]^ Notably, a “boxed warning” was added to promethazine that in children less than 2 years of age, this drug was contraindicated, and for those older than 2 years of age, a strengthened warning was proposed.^[11]^

To the best of our knowledge, our patient is the oldest among the cases in available literatures. In this patient, there were no apparent risk factors. However, cranial magnetic resonance imaging revealed sporadic ischemic lesions in the bilateral periventricular regions and basal ganglia, indicating possible functional disturbances in these brain regions, which may make the patient susceptible to acute dystonic reaction. Of note, the patient was previously prescribed with flupentixol−melitracen and took it for a long period. Flupentixol, a typical antipsychotic of the thioxanthene, is mainly used in schizophrenic individuals as a long-acting preparation. Extrapyramidal side effects of flupentixol treatment include dyskinesia, tremor, parkinsonism, and dystonia. Melitracen is a tricyclic antidepressant, but its pharmacology remains largely unknown. Nowadays, flupentixol and melitracen are no longer prescribed alone in clinical practice, but their fixed-dose combination (Deanxit tablet) is still popular in the Chinese market. As an old antipsychotic, flupentixol was also possibly to elicit acute dystonia. However, the patient had once taken the same dose of flupentixol and discontinued this agent twice, no extrapyramidal side effects were previously observed. It seems unlikely that the acute orofacial dystonia was secondary to the sudden discontinuation of flupentixol−melitracen or the long-term usage of flupentixol.

The common recommended treatment for acute dystonia is the administration of diphenhydramine or benztropine intravenously, both of which are anticholinergic agents.^[12]^ It was a pity that our patient did not try any of the aforementioned anticholinergic agents. Benzodiazepines are second-line choice for patients who did not fully respond to anticholinergic therapy.^[12]^ Indeed, a combination of promethazine−chlordiazepoxide with fixed dose was taken by our patient. Chlordiazepoxide belongs to the benzodiazepine class and has sedative and hypnotic functions. Therefore, it can help to relax skeletal muscle and is unlikely to cause acute dystonia. In addition, local injection of Botulinum toxin is also recommended. In our patient, clonazepam supplement failed to alleviate the dystonic symptoms, which was later improved by Botulinum toxin.

To conclude, we report a case of acute onset of orofacial dystonia following promethazine treatment. The patient was an old woman with ischemic changes in the basal ganglia, which may predispose her to acute dystonic condition. Our case outlines a significant adverse effect of promethazine, which warrants attentions from clinical practitioners when prescribing this medication.

## Acknowledgments

The authors thank the patient and her guardian for their understanding to publish this case study.

## Author contributions

**Data curation:** Ruili Zhang, Jianbo Lai.

**Funding acquisition:** Jianbo Lai.

**Investigation:** Ruili Zhang, Jianbo Lai.

**Writing – original draft:** Ruili Zhang, Jianbo Lai.

**Writing – review & editing:** Jinwen Huang.
